# Association of *Staphylococcus aureus* Bacterial Load and Colonization Sites With the Risk of Postoperative *S. aureus* Infection

**DOI:** 10.1093/ofid/ofae414

**Published:** 2024-07-23

**Authors:** Darren P R Troeman, Derek Hazard, Cornelis H W van Werkhoven, Leen Timbermont, Surbhi Malhotra-Kumar, Martin Wolkewitz, Alexey Ruzin, Frangiscos Sifakis, Stephan Harbarth, Jan A J W Kluytmans, ASPIRE-SSI Study Group, Herman Goossens, Jelle Vlaeminck, Tuba Vilken, Basil Britto Xavier, Christine Lammens, Marc Bonten, Marjolein van Esschoten, Fleur Paling, Claudia Recanatini, Frank Coenjaerts, Brett Selman, Christine Tkaczyk, Susanne Weber, Miquel Ekkelenkamp, Lijckle van der Laan, Bas Vierhout, Elodie Couvé-Deacon, Miruna David, David Chadwick, Martin Llewelyn, Andrew Ustianowski, Tony Bateman, Damian Mawer, Biljana Carevic, Sonja Konstantinovic, Zorana Djordjevic, María Dolores del Toro López, Juan P Horcajada, Dolores Escudero, Miquel Pujol Rojo, Julián de la Torre Cisneros, Francesco Castelli, Giuseppe Nardi, Pamela Barbadoro, Mait Altmets, Piret Mitt, Adrian Todor, Serban Ion Bubenek Turconi, Dan Corneci, Dorel Săndesc, Valeriu Gheorghita, Radim Brat, Ivo Hanke, Jan Neumann, Tomáš Tomáš, Wim Laffut, Annemie Van den Abeele, Sanne Van Rooij, Edith Schasfoort, Curt Brugman, Janet Couperus, Karin Van Beek, Nienke Cuperus, Sophie Corthals, Liesbeth Bryssinck, Stalin Solomon, Sabine Chapelle, Anouk Vanderstraeten

**Affiliations:** Julius Center for Health Sciences and Primary Care, University Medical Center Utrecht, Utrecht University, Utrecht, The Netherlands; Institute of Medical Biometry and Statistics, Faculty of Medicine and Medical Center, University of Freiburg, Freiburg, Germany; Julius Center for Health Sciences and Primary Care, University Medical Center Utrecht, Utrecht University, Utrecht, The Netherlands; Laboratory of Medical Microbiology, Vaccine and Infectious Disease Institute, University of Antwerp, Antwerp, Belgium; Laboratory of Medical Microbiology, Vaccine and Infectious Disease Institute, University of Antwerp, Antwerp, Belgium; Institute of Medical Biometry and Statistics, Faculty of Medicine and Medical Center, University of Freiburg, Freiburg, Germany; Microbial Sciences, R&D BioPharmaceuticals, AstraZeneca, Gaithersburg, Maryland, USA; Gilead Sciences, Inc, Foster City, California, USA; Infection Control Programme and WHO Collaborating Center, Geneva University Hospitals and Faculty of Medicine, Geneva, Switzerland; Julius Center for Health Sciences and Primary Care, University Medical Center Utrecht, Utrecht University, Utrecht, The Netherlands; Department of Medical Microbiology, University Medical Center Utrecht, Utrecht University, Utrecht, The Netherlands

**Keywords:** colonization, nasal carriage, health care–associated infection, surgical site infection, bloodstream infection, hospital-acquired infection, *Staphylococcus aureus*

## Abstract

**Background:**

The independent effects of extranasal-only carriage, carriage at multiple bodily sites, or the bacterial load of colonizing *Staphylococcus aureus* (SA) on the risk of developing SA surgical site infections and postoperative bloodstream infections (SA SSI/BSIs) are unclear. We aimed to quantify these effects in this large prospective cohort study.

**Methods:**

Surgical patients aged 18 years or older were screened for SA carriage in the nose, throat, or perineum within 30 days before surgery. SA carriers and noncarriers were enrolled in a prospective cohort study in a 2:1 ratio. Weighted multivariable Cox proportional hazard models were used to assess the independent associations between different measures of SA carriage and occurrence of SA SSI/BSI within 90 days after surgery.

**Results:**

We enrolled 5004 patients in the study cohort; 3369 (67.3%) were SA carriers. 100 SA SSI/BSI events occurred during follow-up, and 86 (86%) of these events occurred in SA carriers. The number of colonized bodily sites (adjusted hazard ratio [aHR], 3.5–8.5) and an increasing SA bacterial load in the nose (aHR, 1.8–3.4) were associated with increased SA SSI/BSI risk. However, extranasal-only carriage was not independently associated with SA SSI/BSI (aHR, 1.5; 95% CI, 0.9–2.5).

**Conclusions:**

Nasal SA carriage was associated with an increased risk of SA SSI/BSI and accounted for the majority of SA infections. Higher bacterial load, as well as SA colonization at multiple bodily sites, further increased this risk.

Surgical site infections (SSIs) are important complications of surgical procedures. SSIs develop in up to 20% of surgical patients depending on the type of procedure, are associated with longer hospital stays and increased mortality risk (up to 11-fold), and are very costly, with an estimated excess cost of US$20 785 per SSI [1–5]. Postoperative bloodstream infections (BSIs) are also important complications after surgery, though they occur less often than SSIs [6].

Studies have shown that *Staphylococcus aureus* (SA) is an important causative pathogen of postoperative infections, causing ∼30% of documented SSIs [5, 7]. SA is a commensal pathogen that colonizes 20%–30% of the human population at different bodily sites, including the nose, throat, axilla, and perineal region [8, 9]. Earlier studies identified nasal SA carriage as an important risk factor for developing postoperative SA infection [6, 8]. Nasal SA carriage alone has been associated with a 2- to 10-fold increase in the risk of developing SA SSI [10]. However, the impact of extranasal-only carriage, bacterial load, and number of colonized bodily sites on the risk of developing SA SSI or postoperative BSI has been insufficiently investigated.

Therefore, the primary objective of this study was to quantify the association between these measures of SA carriage and SA SSI or postoperative BSI (SA SSI/BSI). In addition, we estimated the proportion of SA SSI/BSIs that could be attributed to preoperative SA carriage. We hypothesized that all measures of endogenous SA carriage would independently be associated with an increased risk of SA SSI/BSI and that there would be a dose–response relationship for number of colonized bodily sites and bacterial load.

## METHODS

### Study Design

We used data from the ASPIRE-SSI study (ClinicalTrials.gov Identifier: NCT02935244) for this analysis. The rationale and design of this study have been described in detail elsewhere [11]. In short, the ASPIRE-SSI was a prospective observational cohort study in which adult surgical patients were included and followed from surgery up to 90 days after surgery to assess the occurrence of and risk factors for SA postoperative infections. The main results from the ASPIRE-SSI study are published elsewhere [12].

### Setting

Patient recruitment took place at 33 hospitals in 10 European countries, distributed across Europe. Recruitment started in December 2016 and ended in September 2019. In December 2019, the last included patient completed follow-up. The concerned institutional review boards or ethics committees approved the study protocol, and all study participants provided written informed consent before study participation.

### Research Objectives

This study aimed to assess whether (1) SA nasal and extranasal-only carriage are independently associated with the risk of developing SA SSI/BSI; (2) an increasing number of bodily sites colonized with SA would increase the risk of developing SA SSI/BSI; (3) the bacterial load of colonizing SA is independently associated with an increased risk of developing SA SSI/BSI.

### Participants

Patients aged ≥18 years and undergoing 11 different surgical procedures provided consent within 30 days before their surgery for participation in the study. Most of these surgical procedures were clean and elective procedures. Important exclusion criteria were concomitant participation in any interventional study of an antistaphylococcal intervention and an active SSI as the reason for undergoing surgery. The participants underwent SA colonization screening in the nose, throat, and perineum. These participants constituted the source population. Based on their preoperative SA screening status, ∼5000 SA carriers and noncarriers were enrolled in the study cohort in a 2:1 ratio. This was the main study population. The rationale for the sample size and other exclusion criteria can be found elsewhere [11].

### Variables and Outcomes

The main exposure variable was preoperative SA colonization (different types and patterns). SA screening samples were processed at local (hospital) laboratories on chromogenic media (Colorex Staph Aureus, Biotrading) using standardized methods. Phenotypic criteria were used to assess the presence or absence of SA in the screening samples (SA grew as pink to mauve colonies on this medium). For each culture sample, the bacterial load of colonizing SA was determined semiquantitatively using the quadrant streaking method [13], and the scoring was classified as follows: NG = no growth; 1+ = light growth; 2 + and 3+ = moderate growth; and 4+ = heavy growth. Based on a literature review, the following variables were considered potential confounders: age, sex, body mass index (BMI; defined as body weight [kg] divided by the square of body height [m^2^]), prior history of SA colonization or infection, preoperative decolonization treatment (if given after SA screening), use of immunosuppressive medication before surgery, Charlson comorbidity index (CCI), American Society of Anesthesiologists physical classification score (ASA score), and site. The main study outcome was the occurrence of SA SSI/BSI within 90 days after surgery. An SA SSI or BSI was defined as the isolation of SA from a surgical wound–related specimen or blood culture, respectively, and fulfilling the criteria for an SSI [14] or BSI (laboratory-confirmed bloodstream infection definition 1) [15] according to the Centers for Disease Control and Prevention guidelines. These clinical samples were processed and analyzed according to local guidelines. Other study outcomes included mortality up to 90 days postsurgery. Data were retrieved at fixed time points during follow-up from the medical records of the participants and by contacting the participants or their next of kin.

## STATISTICAL ANALYSIS

Categorical variables are reported as number (%) and continuous variables as median (interquartile range [IQR]). All study estimates were determined in the study cohort and estimated for the source population using weighting methods. These weighting methods considered the likelihood of participants to be included in the study cohort to determine the study estimates for the source population. A detailed description of these methods is given in the Supplemental Data.

### Risk Factor Analysis

Cox proportional hazard models were used to determine the independent association between the different measures of preoperative SA colonization (at any bodily site, by colonization site, by bacterial load, and by the number of colonized bodily sites) and SA SSI/BSI within 90 days postsurgery.

In 3 analyses (at any bodily site, by colonization site, and by the number of colonized bodily sites), we used propensity scores (PS) to adjust for confounding factors. As the primary exposure (SA colonization) was included as a binary variable in these analyses, PS were used to most effectively reduce the dimensionality of the confounding factors before adjustment [16]. Using logistic regression models, we calculated the propensity of each participant to be preoperatively colonized with SA based on a set of confounders. The PS were then included in the Cox models for confounding adjustment. In these analyses, the comparison group was always the noncarriers, whereas the composition of the carriers differed depending on the specific analysis. The balance of the baseline covariates between the carriers and noncarriers was assessed graphically by stratifying the weighted data based on the quintiles of the PS and then comparing the distribution of the covariates of the carriers vs noncarriers using within-quintile side-by-side boxplots [17].

Regarding the fourth analysis, only the bacterial load of nasal SA had a linear relationship with the incidence of SA SSI/BSI (Supplementary Figure 1). In addition, there were only a few SA SSI/BSI events in extranasal-only SA carriers. Therefore, we only assessed the association between the bacterial load of nasal SA and SA SSI/BSI. The bacterial load of nasal SA was included as a continuous variable in this model, as it had a linear relationship with the Martingale residuals of the null model. We also included a binary variable for nasal SA carriage in the model in order to disentangle the added value of having information about the bacterial load of nasal SA in addition to information about the SA carriage status in the nose. For these reasons, we could not use PS to adjust for confounding. Instead, all confounding variables were included separately in the model. The participants colonized extranasally only comprised the comparison group. For interpretative purposes, we present the combined estimates of nasal SA colonization and bacterial load of nasal SA.

Adjusted hazard ratios (aHRs) are presented with 95% CIs. A 2-sided *P* < .05 was considered significant.

### Missing Values

The percentage of missing values across the variables varied between 0% and 4.0%. A total of 364 (7.3%) records included missing values. Primary reasons for missing data were that the data could not be retrieved from the medical records or that the perineum screening sample was not collected. Missing values were considered missing at random, and multivariable imputation by chained equation (MICE) was used to create 10 imputed data sets for the analyses. Incomplete variables were imputed under fully conditional specifications, using the default settings of the MICE, version 3.14, package [18]. The parameters of interest were estimated in each imputed data set separately and pooled using Rubin's rule [19].

### Calculation of the Population-Attributable Fraction

The population-attributable fraction (PAF) of SA SSI/BSI that could be attributed to SA carriage was estimated in a 3-step procedure. Per imputed data set: (I) 10 000 bootstrap samples were created from the study cohort. (II) Each bootstrap sample was inflated using the weights to recreate the source population (representative sample of the general surgical population), and in each the PAF [20] was determined using the Cox model for the association between SA colonization at any site and SA SSI/BSI. (III) The sequence of 10 000 PAF estimates was used to derive the median PAF estimate with the 95% CI (2.5th and 97.5th percentile) of each imputed data set. Lastly, the 10 PAF estimates and 95% CIs were pooled using Rubin's rule [19].

### Sensitivity Analyses

To assess the robustness and possible effect of imputation on the study results, we repeated the analyses on the subset of complete cases.

All analyses were conducted with SPSS, version 26, and R, version 4.0.2.

## RESULTS

### Patient Flow and Characteristics

**Figure 1.**
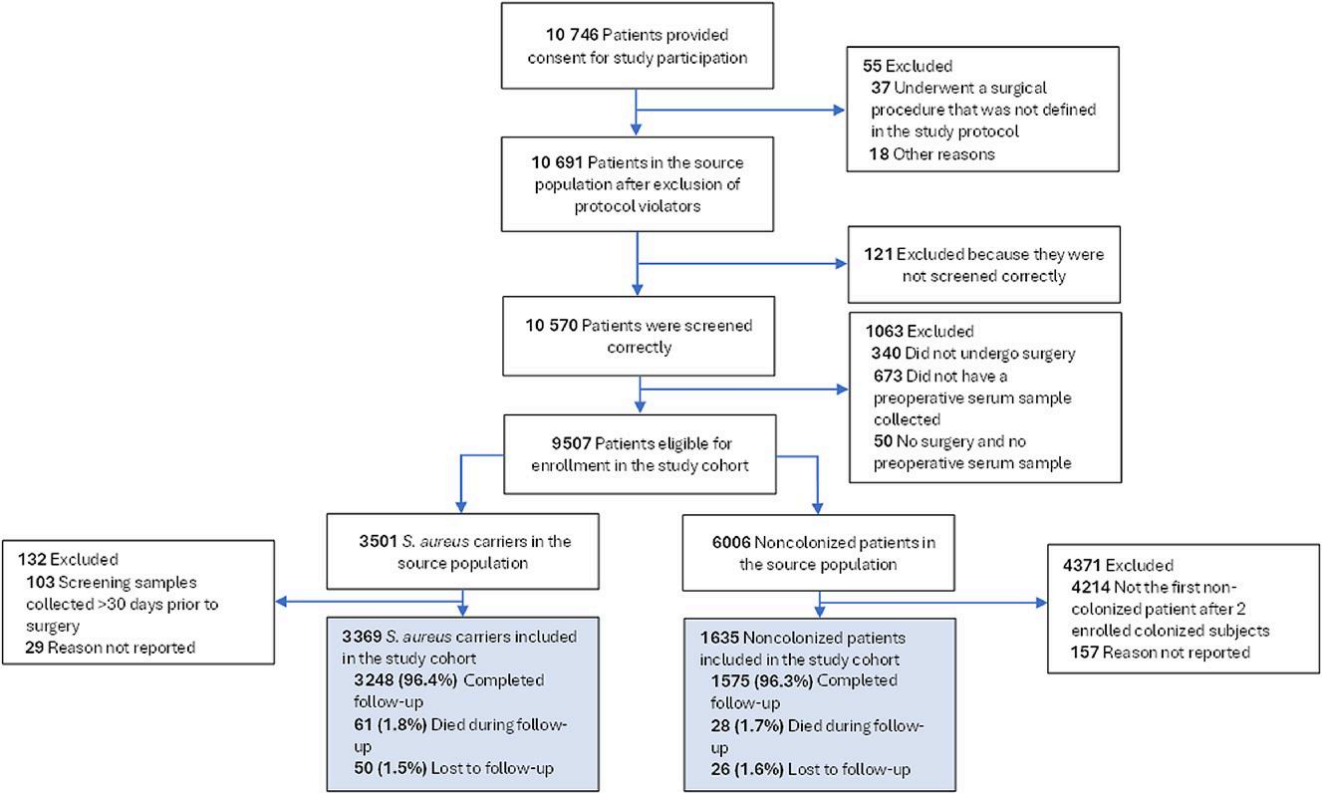
Flow of subjects in the ASPIRE-SSI study. The analysis population is indicated by the shaded boxes.

A total of 10 691 patients were included in the source population. Most of them (44%) were recruited in Southern Europe (Supplementary Table 1). Five thousand four source population patients were included in the study cohort, of whom 3369 (67.3%) were colonized with SA preoperatively (Figure 1). The baseline characteristics of the study cohort are presented in Table 1. Most patients underwent open cardiac, knee, or hip prosthesis surgery, 2494 (49.8%) patients were male, and the median (IQR) age was 66 (56–72) years. Except for preoperative decolonization treatment and history of SA colonization or infection, the baseline characteristics of the SA carriers and noncarriers were comparable. A total of 89 (1.8%) patients died during follow-up.

**Table 1.** Characteristics of the Study Cohort

Characteristic	With SA Carriage, No. (%) (n = 3369)	Without SA Carriage, No. (%) (n = 1635)
Type of surgery	…	…
Craniotomy	198 (5.9)	92 (5.6)
Laminectomy	307 (9.1)	151 (9.2)
Spinal fusion	107 (3.2)	44 (2.7)
Central artery reconstructive surgery	99 (2.9)	44 (2.7)
Peripheral artery bypass surgery	199 (5.9)	97 (5.9)
Mastectomy	317 (9.4)	152 (9.3)
Open cardiac surgery	649 (19.3)	320 (19.6)
Implantable cardioverter defibrillator	55 (1.6)	27 (1.7)
implantation		
Emergency surgery	207 (6.1)	99 (6.1)
Hip prosthesis surgery	577 (17.1)	280 (17.1)
Knee prosthesis surgery	654 (19.4)	329 (20.1)
Median age (IQR), y	65 (55–72)	67 (58–73)
Sex	…	…
Male	1727 (51.3)	767 (46.9)
Female	1642 (48.7)	868 (53.1)
Prior history of SA carriage or infection (including MRSA)	…	…
Yes	329 (9.8)	43 (2.6)
No	3033 (90)	1589 (97.2)
Missing	7 (0.2)	3 (0.2)
Bodily site of preoperative SA carriage^a^	…	…
Nose only	1773 (16.8)	NA
Throat only	531 (5.0)	NA
Perineum only	230 (2.2)	NA
Nose and throat	690 (6.5)	NA
Nose and perineum	248 (2.3)	NA
Throat and perineum	54 (0.5)	NA
Nose, throat, and perineum	199 (1.9)	NA
Current MRSA carriage	…	…
Yes	127 (3.8)	NA
No	3011 (89.4)	NA
Missing	231 (6.9)	NA
BMI (IQR), kg/m^2^	27.9 (24.9–31.6)	27.7 (24.8–31.1)
Missing	44 (1.3)	23 (1.4)
American Society of Anesthesiologists physical classification status	…	…
Class I	326 (10.1)	136 (8.6)
Class II	1445 (44.8)	707 (44.9)
Class III	1269 (39.3)	640 (40.6)
Class IV	184 (5.7)	93 (5.9)
Class V	3 (0.1)	0 (0)
Missing	142 (4.2)	59 (3.6)
Median Charlson comorbidity index (IQR)	1 (0–2)	1 (0–2)
Missing	1 (0)	2 (0.1)
Receipt of immunosuppressive medication within 2 wk of surgery	…	…
Yes	171 (5.1)	75 (4.6)
No	3194 (94.8)	1559 (95.4)
Missing	4 (0.1)	1 (0.1)
Receipt of decolonization treatment	…	…
Yes	831 (24.7)	282 (17.20)
No	2538 (75.3)	1353 (82.8)

Abbreviations: IQR, interquartile range; MRSA, methicillin-resistant *S. aureus*; NA, not applicable; SA, *S. aureus*.

^a^Includes results from 84 emergency surgery patients from whom only nose and throat screening samples were collected. Of these patients, 46 were nose-only carriers, 14 were throat-only carriers, and 24 were nose and throat carriers. Emergency patients could be enrolled in the study if at least 2 body sites, including the nose, were screened for *S. aureus* colonization before surgery.

### Preoperative *Stapylococcus aureus* Colonization

A total of 10 570 source population patients were screened for preoperative SA colonization according to protocol (Figure 1). Overall, 3725 (35.2%) patients were colonized with SA before surgery. The prevalence of preoperative SA colonization at different bodily sites in the source population is presented in Table 1. There were important differences in the prevalence of preoperative SA carriage between surgery types (Supplementary Table 2) and European regions (Supplementary Table 1).

### *Staphylococcus aureus* Outcomes

A total of 100 SA SSI/BSIs occurred in the study cohort (91 SA SSIs, 4 SA BSIs, and 5 SSIs with secondary BSI). Eighty-six of these SA SSI/BSIs occurred in SA carriers. After weighting, the weighted study cohort consisted of 9657 patients, of whom 119 (1.2%) developed an SA SSI/BSI. The weighted 90-day cumulative incidence of SA SSI/BSI, in aggregate and stratified by preoperative SA colonization status, is presented in Table 2. Unweighted incidence estimates are provided in Supplementary Table 3.

**Table 2.** Weighted Cumulative Incidence of SA SSI/BSI

Population	No. of Subjects	No. of SA SSI/BSI Events	Cumulative Incidence per 100 Subjects, Median (95% CI)^a^	Time to SA SSI/BSI (After Surgery), Median (IQR), d
SA carriers (any bodily site)	3369	86	2.6 (2.1–3.1)	19 (13–33)
Noncarriers	6288	33	0.5 (0.2–0.9)	22 (11–28)
All subjects	9657	119	1.2 (1.0–1.6)	20 (13–33)

Abbreviations: BSI, bloodstream infection; IQR, interquartile range; SA, *S. aureus*; SSI, surgical site infection.

^a^Ninety-five percent CIs were bootstrapped. Ten thousand bootstrap samples of the study cohort were made, after which the bootstrap samples were inflated using the weights. In each inflated bootstrap sample, the cumulative incidence was calculated. The sequence of 10 000 cumulative incidences were used to derive the median cumulative incidence with 95% CI.

### *Staphylococcus aureus* Colonization and SSI/BSI

The association between SA carriage at different bodily sites and SA SSI/BSI, as well as the estimated PAFs, is presented in Table 3. Both SA carriage at any site (aHR, 4.6; 95% CI, 2.1–10.0) and nasal carriage (aHR, 4.2, 95% CI, 2.0–8.6) were independently associated with an increased risk of developing SA SSI/BSI within 90 days after surgery. The PAFs for SA carriage at any site and nasal carriage were, respectively, 56.8% (95% CI, 34.8%–78.7%) and 48.8% (95% CI, 29.7%–67.9%). Conversely, extranasal-only carriage was not an independent risk factor. The risk of developing a postoperative SA infection increased as the number of preoperatively colonized bodily sites increased (aHR increased from 3.5 to 8.5 as the number of colonized bodily sites increased from 1 to 3) (Table 4). Also, per 1-unit incremental increase in the bacterial load in the nose (range from no growth to 4+ growth), the aHR for developing SA SSI/BSI increased 1.23-fold (95% CI, 1.05–1.43) (Table 5). Comparable results were obtained when the analyses were restricted to complete cases only (Supplementary Tables 4–6). Additionally, the median nasal SA bacterial load increased as the number of colonized bodily sites increased (Supplementary Table 7). We could not conduct any meaningful subgroup analysis for the association between MRSA carriage and MRSA SSI/BSI as only 3.8% (127/3369) of carriers were MRSA colonized, and we only documented 4 cases of MRSA SSI/BSI.

**Table 3.** Multivariable Weighted Analysis of the Association Between Preoperative SA Carriage and SA SSI/BSI Within 90 Days After Surgery and Corresponding Population-Attributable Fraction

Bodily Site of *S. aureus* Colonization	No. of SA SSI/BSI Events/No. of Subjects (%)	Adjusted HR (95% CI)	PAF, Median (95% CI), %
Model 1	…	…	…
Noncarriers	14/1631 (0.9)	Reference	Reference
Carriage at any bodily site	86/3373 (2.6)	4.6 (2.1–10.0)	56.8 (34.8–78.7)
Model 2	…	…	…
Noncarriers	14/1631 (0.9)	Reference	Reference
Carriage in the nose	76/2615 (2.9)	4.2 (2.0–8.6)	48.8 (29.7–67.9)
Model 3	…	…	…
Noncarriers	14/1631 (0.9)	Reference	Reference
Extranasal-only carriage	10/758 (1.3)	1.5 (0.9–2.5)	12.1 (−0.01 to 25.5)

Results are based on 10 imputed data sets. Depicted above is the average number of SA SSI/BSI events for carriers and noncarriers over the imputed data sets. As the perineum sample was also imputed for the patients who did not have that screening sample collected, the total numbers of carriers and noncarriers are slightly different from the numbers presented in the flowchart. Model 1 was adjusted for age, sex, BMI, history of SA infection or colonization, use of immunosuppressive medication, Charlson comorbidity index, ASA score, preoperative decolonization, and site. Model 2 was additionally adjusted for extranasal SA carriage, compared with model 1. Model 2 also includes patients colonized at multiple bodily sites, including the nose. Model 3 was additionally adjusted for nasal *S. aureus* carriage, compared with model 1.

Abbreviations: ASA, American Society of Anesthesiologists physical classification score; BMI, body mass index; BSI, bloodstream infection; HR, hazard ratio; IQR, interquartile range; PAF, population-attributable fraction; SA, *S. aureus*; SSI, surgical site infection.

**Table 4.** Multivariable Weighted Analysis of the Association Between the Number of SA Colonized Body Sites and the Risk of SA SSI/BSI Within 90 Days After Surgery

No. of SA Colonized Body Sites	No. of SA SSI/BSI Events/No. of Subjects (%)	Adjusted HR (95% CI)^a^
Noncarriers	14/1631 (0.9)	Reference
Carriage at 1 bodily site	53/2309 (2.3)	3.5 (1.7–7.2)
Carriage at 2 bodily sites	24/891 (2.7)	5.3 (2.1–13.4)
Carriage at 3 bodily sites	9/174 (5.2)	8.5 (2.2–33.8)

Results based on 10 imputed data sets. Depicted above is the average number of SA SSI/BSI events for carriers and noncarriers over the imputed data sets. As the perineum sample was also imputed for the patients who did not have that screening sample taken, the total numbers of carriers and noncarriers are slightly different from the numbers presented in the flowchart.

Abbreviations: BSI, bloodstream infection; HR, hazard ratio; IQR, interquartile range; SA, *S. aureus*; SSI, surgical site infection.

^a^Adjusted for: age, sex, BMI, history of SA infection or colonization, use of immunosuppressive medication, Charlson comorbidity index, ASA score, preoperative decolonization, and hospital site.

**Table 5.** Association Between Bacterial Load of Colonizing SA in the Nose and SA SSI/BSI Within 90 Days After Surgery

SA Carriage Status in the Nose and Semiquantitative Bacterial Load of Colonizing SA	Adjusted HR (95% CI)^a^
Noncolonized in the nose, but colonized extranasally	Reference
Carriage of 1+ bacterial load of SA	1.8 (1.0–2.7)
Carriage of 2+ bacterial load of SA	2.3 (1.4–3.1)
Carriage of 3+ bacterial load of SA	2.8 (1.9–3.6)
Carriage of 4+ bacterial load of SA	3.4 (2.5–4.3)

Results based on 10 imputed data sets. Depicted above is the average number of SA SSI/BSI events for carriers and noncarriers over the imputed data sets. The weighted analysis included only colonized patients. The patients colonized extranasally only served as the reference group.

Abbreviations: BSI, bloodstream infection; HR, hazard ratio; SA, *S. aureus*; SSI, surgical site infection.

^a^Adjusted for: SA throat colonization, SA perineum colonization, age, sex, body mass index, history of SA infection or colonization, use of immunosuppressive medication, Charlson comorbidity index, American Society of Anesthesiologists score, preoperative decolonization, and number of colonized bodily sites. Hospital site was used as the cluster term.

## DISCUSSION

In this large cohort study, the prevalence of SA colonization at any bodily site was 35%. Endogenous SA carriage at any bodily site, and particularly in the nose, was independently associated with an increased risk of developing SA SSI/BSI. In addition, we observed a linear relationship between the semiquantitative bacterial load of SA in the nose and the occurrence of SA SSI/BSI. Lastly, we found that the risk for developing SA SSI/BSI increased as the number of preoperatively colonized bodily sites increased.

Previous studies have shown that the nose is the primary ecological niche for SA carriage and that nasal SA carriage is an independent risk factor for SA infection [8, 21]. Our results are in line with these findings. However, SA carriage at any bodily site was also independently associated with an increased risk of developing SA SSI/BSI. This finding was not surprising, as SA nasal carriage was highly prevalent within the carriers of our study. The importance of SA carriage in the etiology of postoperative SA infection was also evident from the PAF, as >50% of cases could have been prevented if endogenous SA carriage would have been fully eradicated. In contrast, SA extranasal-only carriage was not independently associated with an increased risk of SA SSI/BSI.

Despite this, the risk of developing SA SSI/BSI seemed to increase as the number of preoperatively colonized bodily sites increased. Based on our data, we argue that (I) this could be a reflection of the increasing prevalence of nasal carriage as the number of colonized bodily sites increased (+/−70%, +/−95%, and 100% nasal carriage in carriers colonized at only 1, 2, or 3 bodily sites) and (II) patients who were colonized at more bodily sites had a higher “average” bacterial load of colonizing SA, and therefore a higher risk of infection with SA (as shown in the Supplementary Data). Additionally, our results suggest that the bacterial load of colonizing SA is also important when assessing the risk of developing SA infection. This concept has previously been described [9].

The screening of multiple bodily sites for SA carriage may be important when the aim is to identify the patients most at risk of SA SSI/BSI as a small but possibly relevant effect of extranasal-only carriage cannot be excluded based on these data. Furthermore, depending on the resources at hand, a “screen-and-treat” strategy consisting of screening multiple bodily sites for SA colonization and then decolonizing the carriers could be a cost-effective strategy for preventing SA SSI [22]. Based on this, we argue that all screen-and-treat SA decolonization strategies should include nasal SA screening. If feasible, the screening of additional bodily sites could be considered.

Lastly, to our knowledge, only 1 earlier study assessed the effect of extranasal SA carriage and the risk of SA infection. One small study of hemodialysis patients suggested that extranasal SA carriage was equally relevant as a risk factor for catheter-related staphylococcal infections as nasal SA carriage [23]. However, the investigators did not adjust for confounding factors. For this reason, the results of this study should be interpreted with caution.

The present study has several strengths. This was the largest prospective SA carriage study in surgical patients. The prospective recruitment of a very large number of patients from different countries and geographical areas based on clear and relevant criteria was a major strength of this study. Other strengths were that we standardized the methods for SA screening across participating sites and that we used widely accepted criteria to determine the study outcomes. We also aimed for a wide applicability of the study results by including patients undergoing several types of surgical procedures. A limitation of this study was that we could not conduct a meaningful subgroup analysis of the relationship between MRSA carriage and MRSA SSI/BSI because of the low number of MRSA carriage and MRSA SSI/BSI events in the study. Another limitation was that although all sites made use of a uniform culture method to assess SA colonization, the evaluation was only based on colonial morphology; no additional microbiological confirmation was requested. As the culture method did not have perfect sensitivity and specificity, this could have led to misclassification of the SA carriage status for some patients [24].

## CONCLUSIONS

Preoperative SA carriage, especially when present in the nose, is independently associated with SA SSI/BSI and accounted for more than half of the cases. In addition, the bacterial load of nasal SA, as well as the number of SA colonized bodily sites, is also important and should be taken into account when assessing the risk of developing SA SSI/BSI. Our results underscore the importance of preoperative SA carriage in the etiology of postoperative SA infections and provide evidence in support of SA decolonization strategies aimed at preventing SA infections. Such interventions have been shown to be efficacious, effective [25, 26], and cost-effective [27].

## Supplementary Material

ofae414_Supplementary_Data

## Supplementary Data

Supplementary materials are available at *Open Forum Infectious Diseases* online. Consisting of data provided by the authors to benefit the reader, the posted materials are not copyedited and are the sole responsibility of the authors, so questions or comments should be addressed to the corresponding author.
