# Supplementary material for: Association of Staphylococcus aureus Bacterial Load and Colonization Sites With the Risk of Postoperative S. aureus Infection
Source: Open Forum Infect Dis. 2024 Jul 23;11(8):ofae414. doi: 10.1093/ofid/ofae414 (PMC11304588; doi:10.1093/ofid/ofae414)
Supplement: ofae414_Supplementary_Data [file ofae414_supplementary_data.docx]

SUPPLEMENTAL MATERIAL

**Manuscript title: *Staphylococcus aureus* colonization as a risk factor for SA surgical site infections and postoperative bloodstream infections: secondary analyses from a prospective multicenter observational study**

**Contents**

[Supplementary table 1. Number of included subjects per European subregion 3](#_Toc120877856)

[Supplementary table 2. Weighted prevalence of preoperative SA carriage at any body site by surgery type 4](#_Toc120877857)

[Supplementary table 3. Unweighted cumulative incidence of SA SSI and postoperative BSI within 90 days post-surgery 5](#_Toc120877858)

[Supplemental table 4. Multivariable analysis to assess the association between preoperative SA carriage and SA SSI/BSI within 90 days post-surgery and estimate the fraction of the SA SSI/BSI that is attributed to SA carriage (complete case analysis) 6](#_Toc120877859)

[Supplementary table 5. Multivariable analysis to assess the association between the number of colonized body sites and SA SSI/BSI within 90 days post-surgery (complete case analysis) 7](#_Toc120877860)

[Supplemental table 6. Association between the bacterial load of colonizing SA in the nose and SA SSI/BSI within 90 days post-surgery (complete case analysis) 8](#_Toc120877861)

[Supplemental table 7. The distribution of the semi-quantitative bacterial load of SA in the nose, stratified by the number of preoperatively colonized body sites 9](#_Toc120877862)

[Supplemental figure 1. Graphic representation of the relationship between the semi-quantitative bacterial load of SA in the nose, throat, or perineum, and the incidence of SA SSI or postoperative BSI within 90 days post-surgery. 10](#_Toc120877863)

**Description of the weighting methods**

As reported in the manuscript, SA carriers and non-carriers were enrolled into the study cohort in a 2:1 ratio. However, this ratio was approximately 1:3 in the overarching source population. Because the source population was a random sample of the general population, it was the population that we were interested in making inference on. For this reason we aimed to recreate the source population by weighting the study cohort subjects with the inverse probability of their inclusion in the study cohort [1]. After conducting multiple imputation for missing values in the source population, we fitted a logistic regression model using predictor variables available for the source population, to estimate the probability of inclusion. After this, we took the inverse of this probability as the weight. The predictor variables are listed in the table below, as well as their distributions in the source population, study cohort, and weighted study population. There is good agreement between the source and weighted population. The calculated weights were used for the incidence calculations and in the risk factor analysis in the accompanying manuscript.

| **Characteristic** | **Level** | **Source population (correctly screened)** | **Study cohort** | **Weighted population** |
| --- | --- | --- | --- | --- |
| N |  | 10570 | 5004 | 9657 |
| SA colonization (%) | Yes  No | 3725 (35.2)  6845 (64.8) | 3369 (67.3)  1635 (32.7) | 3369 (34.9)  6288 (65.1) |
| Region (%) | North | 2171 (20.5) | 912 (18.2) | 2327 (24.1) |
|  | East | 1232 (11.7) | 502 (10.0) | 1160 (12) |
|  | South | 4679 (44.3) | 2371 (47.4) | 4180 (43.3) |
|  | West | 2488 (23.5) | 1219 (24.4) | 1990 (20.6) |
| Year of inclusion (%) | 2016 | 3 (0.0) | 1 (0.0) | 3 (0.0) |
|  | 2017 | 1173 (11.1) | 505 (10.1) | 858 (8.9) |
|  | 2018 | 5537 (52.4) | 2743 (54.8) | 4952 (51.3) |
|  | 2019 | 3857 (36.5) | 1755 (35.1) | 3844 (39.8) |
| Age (median [IQR]) |  | 66 [57;73] | 66 [56; 72] | 66 [56;73] |
| Sex (%) | Female | 5401 (51.1) | 2510 (50.2) | 5113 (52.9) |
|  | Male | 5166 (48.9) | 2494 (49.8) | 4544 (47.1) |
|  | Missing | 3 (0.0) | 0 | 0 |
| BMI (median[IQR]) |  | 27.8 [24.8;31.5] | 27.8 [24.8; 31.3] | 27.8[24.9;31.3] |
| ASA score (%) | 1 | 926 (8.8) | 462 (9.2) | 955 (9.9) |
|  | 2 | 4331 (41) | 2152 (43) | 4318 (44.7) |
|  | 3 | 4093 (38.7) | 1909 (38.1) | 3400 (35.2) |
|  | ≥4 | 553 (5.2) | 280 (5.6) | 500 (5.2) |
|  | Missing | 667 (6.3) | 201 (4.0) | 484 (5) |
| Non-removable implant present prior to surgery (%) | Yes | 2549 (24.1) | 1207 (24.1) | 2398 (24.8) |
|  | No | 8003 (75.7) | 3790 (75.8) | 7242 (75.0) |
|  | Missing | 18 (0.2) | 7 (0.1) | 17 (0.2) |
| Diabetes mellitus (%) | Yes | 1971 (18.6) | 974 (19.5) | 1739 (18) |
|  | No | 8588 (81.3) | 4030 (80.5) | 7918 (82) |
|  | Missing | 11 (0.1) | 0 | 0 |
| Type of surgery | Open heart surgery | 2316 (21.9) | 969 (19.4) | 1772 (18.3) |
|  | Implantable cardioverter defibrillator implantation | 175 (1.7) | 82 (1.6) | 219 (2.3) |
|  | Knee prosthesis surgery | 1919 (18.2) | 983 (19.6) | 1895 (19.6) |
|  | Hip prosthesis surgery | 1669 (15.8) | 857 (17.1) | 1523 (15.8) |
|  | Laminectomy | 953 (9.0) | 458 (9.2) | 925 (9.6) |
|  | Spinal fusion surgery | 371 (3.5) | 151 (3.0) | 446 (4.6) |
|  | Peripheral artery bypass surgery | 618 (5.8) | 296 (5.9) | 438 (4.5) |
|  | Central artery reconstruction surgery | 255 (2.4) | 143 (2.9) | 189 (2.0) |
|  | Mastectomy | 1079 (10.2) | 469 (9.4) | 1031 (10.7) |
|  | Craniotomy | 534 (5.1) | 290 (5.8) | 519 (5.4) |
|  | Emergency surgery | 681 (6.4) | 306 (6.1) | 700 (7.2) |
| Serum sample collected (%) | Yes | 9847 (93.2) | 5004 (100) | 9657 (100) |
|  | No | 723 (6.8) | 0 | 0 |
| Planned surgery occurred | Yes | 10180 (96.3) | 5004 (100) | 9657 (100) |
|  | No | 390 (3.7) | 0 | 0 |

Abbreviations: ASA. American Society of Anesthesiologist’s; BMI, body mass, index; IQR, interquartile range.

| **Supplementary table 1. Number of included subjects per European subregion** | | | | | |
| --- | --- | --- | --- | --- | --- |
| **European sub-region** | **No. of participating hospitals** | **Participating countries** | **No. of included source population subjects (%)** | **No. of included study population subjects (%)** | **Prevalence of SA colonization at any body site (%) in the source population** |
| North | 8 | Estonia | 427 (4.0%) | 177 (3.5%) | 30.2% |
|  |  | United Kingdom | 1744 (16.5%) | 735 (14.7%) | 37.5% |
| East | 8 | Czech Republic | 857 (8.1%) | 373 (7. 5%) | 30.2% |
|  |  | Romania | 375 (3.6%) | 129 (2.6%) | 24% |
| South | 11 | Italy | 1038 (9.8%) | 424 (8.5%) | 30.8% |
|  |  | Serbia | 1489 (14.0%) | 1166 (23.3%) | 55.5% |
|  |  | Spain | 2152 (20.4%) | 781 (15.6%) | 24.9% |
| West | 6 | Belgium | 122 (1.2%) | 57 (1.1%) | 36.9% |
|  |  | France | 778 (7.4%) | 348 (7.0%) | 30.7% |
|  |  | The Netherlands | 1588 (15.0%) | 814 (16.3%) | 39.4% |

Abbreviations: No., number; SA, *S. aureus*.

| **Supplementary table 2. Weighted prevalence of preoperative SA carriage at any body site by surgery type** | | |
| --- | --- | --- |
| **Type of surgery** | **No. of subjects** | **Prevalence of SA carriage at any body site,**  **Median % (95% CI)** |
| Cardiovascular surgery | 2618 | 38.8% (36.1%; 41.8%) |
| Orthopedic surgery | 3418 | 36.0% (33.5%; 38.8%) |
| Neurosurgery | 1890 | 35.7% (32.1%; 39.6%) |
| Emergency surgery | 700 | 30.2% (24.9%; 36.9%) |
| Mastectomy | 1031 | 31.7% (27.5%; 36.2%) |

The prevalence of preoperative SA carriage by surgery type was bootstrapped. 10,000 bootstrap samples of the study cohort were made, after which the bootstrap samples were inflated using the weights. In each inflated bootstrap sample, the prevalence of preoperative SA carriage by surgery type was calculated. The sequence of 10,000 SA carriage prevalences per surgery type was used to derive the median preoperative SA carriage prevalence with 95% CI (2.5^th^ and 97.5^th^ percentile) per surgery type.

Abbreviations: CI, confidence interval; No., number*;* SA, *S. aureus*.

| **Supplementary table 3. Unweighted cumulative incidence of SA SSI and postoperative BSI within 90 days post-surgery** | | | | |
| --- | --- | --- | --- | --- |
| **Population** | **No. of subjects** | **No. of SA SSI or BSI events** | **Cumulative incidence per 100 patients, median (95% CI)^a^** | **Time to SA SSI/BSI in days (from day of surgery), median (IQR)** |
| SA carriers (any location) | 3369 | 86 | 2.6 (2.0; 3.1) | 19 (13-33) |
| Non-carriers | 1635 | 14 | 0.9 (0.4; 1.3) | 22 (13-32.5) |

^a^ 95% CIs were bootstrapped. 10,000 bootstrap samples of the study cohort were made. In each bootstrap sample, the cumulative incidence was calculated. The sequence of 10,000 cumulative incidences was then used to derive the median cumulative incidence with 95% CI.

Abbreviations: BSI, bloodstream infection; CI, confidence interval; IQR, interquartile range; No., number; SA, *S. aureus*; SSI, surgical site infection.

| **Supplemental table 4. Multivariable analysis to assess the association between preoperative SA carriage and SA SSI/BSI within 90 days post-surgery and estimate the fraction of the SA SSI/BSI that is attributed to SA carriage (complete case analysis)** | | |
| --- | --- | --- |
| **Body site of SA colonization** | **Adjusted HR (95% CI)** | **PAF, median % (95% CI)** |
| *Model 1* |  |  |
| Non-carriers | Reference | Reference |
| Carriage at any body site | 4.5 (2.0; 9.8) | 55.5% (33.2; 78.0%) |
| *Model 2* |  |  |
| Non-carriers | Reference | Reference |
| Carriage in the nose | 3.8 (1.7; 8.4) | 55.8% (34.0; 78.0%) |
| *Model 3* |  |  |
| Non-carriers | Reference | Reference |
| Extranasal only carriage | 1.7 (0.7; 4.3) | 8.8% (-0.1; 33.3%) |

Model 1 was adjusted for: age, sex, BMI, history of SA infection of colonization, use of immunosuppressive medication, Charlson comorbidity index, ASA score, preoperative decolonization, site.

Model 2 was additionally adjusted for extranasal SA colonization, compared to model 1. Model 2 also includes patients colonized at multiple body sites, including the nose.

Model 3 was additionally adjusted for nasal *S. aureus* colonization, compared to model 1.

Abbreviations: BSI, bloodstream infection; CI, confidence interval; HR, hazard ratio; IQR, interquartile range; No., number; PAF, population attributable fraction; SA, *S. aureus*; SSI, surgical site infection.

| **Supplementary table 5. Multivariable analysis to assess the association between the number of colonized body sites and SA SSI/BSI within 90 days post-surgery (complete case analysis)** | |
| --- | --- |
| **Number of SA colonized body sites** | **Adjusted HR (95% CI)^a^** |
| Non-carriers | Reference |
| Carriage at one body site | 3.3 (1.6; 6.8) |
| Carriage at two body sites | 4.8 (1.9; 12.6) |
| Carriage at three body sites | 8.8 (2.3; 33.4) |

^a^ Controlled for: age, sex, BMI, history of S. aureus infection of colonization, use of immunosuppressive medication, Charlson comorbidity index, ASA score, preoperative decolonization, site

Abbreviations: BSI, bloodstream infection; CI, confidence intervals; HR, hazard ratio; IQR, interquartile range; No., number; SA, *S. aureus*; SSI, surgical site infection.

| **Supplemental table 6. Association between the bacterial load of colonizing SA in the nose and SA SSI/BSI within 90 days post-surgery (complete case analysis)** | |
| --- | --- |
| **SA colonization status in the nose + Semi-quantitative bacterial load of the colonizing SA** | **Adjusted HR (95% CI)^a^** |
| Non-carriers in the nose, but colonized extranasally | Reference |
| Carriage of 1+ bacterial load of SA | 1.9 (1.0; 2.7) |
| Carriage of 2+ bacterial load of SA | 2.3 (1.5; 3.1) |
| Carriage of 3+ bacterial load of SA | 2.9 (2.1; 3.7) |
| Carriage of 4+ bacterial load of SA | 3.6 (2.8; 4.5) |

Results based on 10 imputed datasets. Depicted above is the average number of SA SSI/BSI events for carriers and non-carriers over the imputed datasets. The analysis included only colonized patients. The patients colonized extranasally only served as the reference group.

^a^ Adjusted for: SA throat colonization, SA perineum colonization, age, sex, body mass index, history of SA infection of colonization, use of immunosuppressive medication, Charlson comorbidity index, American Society of Anaesthesiology score, preoperative decolonization. Site was used as cluster term.

Abbreviations: BSI, bloodstream infection; CI, confidence intervals; HR, hazard ratio; SA, *S. aureus*; SSI, surgical site infection .

| **Supplemental table 7. The distribution of the semi-quantitative bacterial load of SA in the nose, stratified by the number of preoperatively colonized body sites** | | | | | | | |
| --- | --- | --- | --- | --- | --- | --- | --- |
| **Number of SA colonized body sites** | **No. of subjects** | **Number (row %) of patients within each category of bacterial load of SA in the nose** | | | | | **Median bacterial load in the nose** |
|  |  | No Growth | + | ++ | +++ | ++++ |  |
| 0 | 1635 | 1635 (100%) | 0 | 0 | 0 | 0 | No SA present |
| 1 | 2313 | 705 (30.5%) | 631 (27.3%) | 467 (20.2%) | 285 (12.3%) | 225 (9.7%) | + |
| 2 | 887 | 49 (5.5%) | 294 (33.1%) | 220 (24.8%) | 174 (19.6%) | 150 (16.9%) | ++ |
| 3 | 169 | 0 (0.0%) | 34 (20.1%) | 48 (28.4%) | 45 (26.6%) | 42 (24.9%) | +++ |

Abbreviations: No, number; SA, *S. aureus*.

| 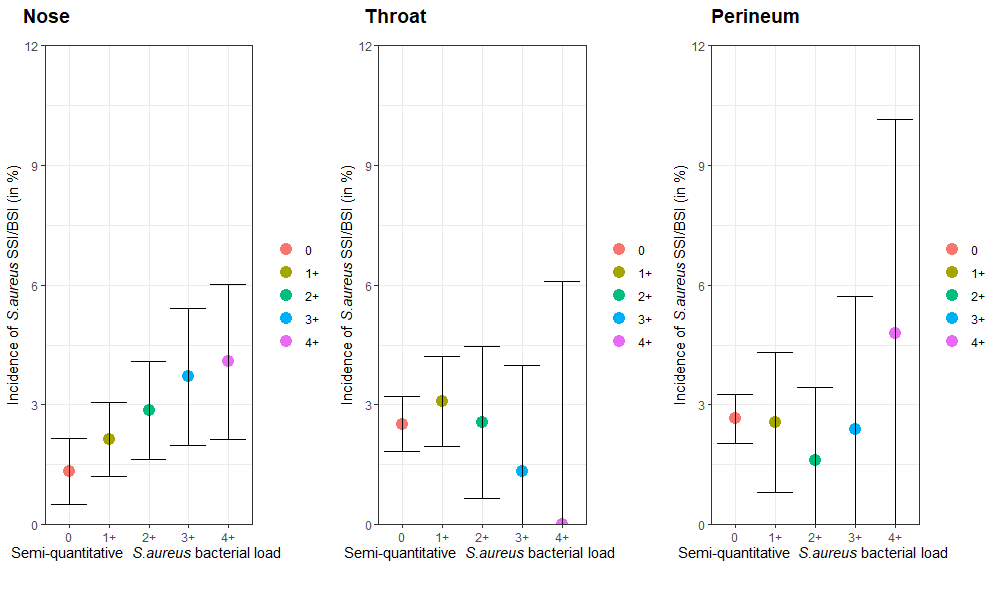 |
| --- |
| **Supplemental figure 1.** **Graphic representation of the relationship between the semi-quantitative bacterial load of SA in the nose, throat, or perineum, and the incidence of SA SSI/BSI within 90 days post-surgery.** |

The colored dots indicate the incidence of the SA SSI/BSI associated with a particular semi-quantitative bacterial load of SA at the respective body site, and the error bars indicate the 95% confidence intervals.

Abbreviations: BSI, bloodstream infection; SA, *S. aureus*; SSI, surgical site infection.

**References**

1. Lee ES, Forthofer RR. Analyzing complex survey data. 2nd edition ed. Vol. 71. SAGE Publications, Inc, **2005**.
